# Anthraquinone Derivatives as an Immune Booster and their Therapeutic Option Against COVID-19

**DOI:** 10.1007/s13659-020-00260-2

**Published:** 2020-08-08

**Authors:** Pukar Khanal, B. M. Patil, Jagdish Chand, Yasmin Naaz

**Affiliations:** 1 Department of Pharmacology and Toxicology, KLE College of Pharmacy, Belagavi, KLE Academy of Higher Education and Research (KAHER), Belagavi, 590010 India; 2 Department of Pharmaceutical Chemistry, KLE College of Pharmacy, Belagavi, KLE Academy of Higher Education and Research (KAHER), Belagavi, 590010 India

**Keywords:** 3CLpro, Anthroquine derivatives, Coronavirus, COVID-19, Immune boost

## Abstract

**Abstract:**

Anthraquinone derivatives are identified for their immune-boosting, anti-inflammatory, and anti-viral efficacy. Hence, the present study aimed to investigate the reported anthraquinone derivatives as immune booster molecules in COVID-19 infection and evaluate their binding affinity with three reported targets of novel coronavirus i.e. 3C-like protease, papain-like protease, and spike protein. The reported anthraquinone derivatives were retrieved from an open-source database and filtered based on a positive druglikeness score. Compounds with positive druglikeness scores were predicted for their targets using DIGEP-Pred and the interaction among modulated proteins was evaluated using STRING. Further, the associated pathways were recorded concerning the Kyoto Encyclopedia of Genes and Genomes pathway database. Finally, the docking was performed using autodock4 to identify the binding efficacy of anthraquinone derivatives with 3C-like protease, papain-like protease, and spike protein. After docking the pose of ligand scoring minimum binding energy was chosen to visualize the ligand–protein interaction. Among 101 bioactives, 36 scored positive druglikeness score and regulated multiple pathways concerned with immune modulation and (non-) infectious diseases. Similarly, docking study revealed torososide B to possess the highest binding affinity with papain-like protease and 3C-like protease and 1,3,6-trihydroxy-2-methyl-9,10-anthraquinone-3-*O*-(6′-*O*-acetyl)-*β*-d-xylopyranosyl-(1 → 2)-*β*-d-glucopyranoside with spike protein.

**Graphic Abstract:**

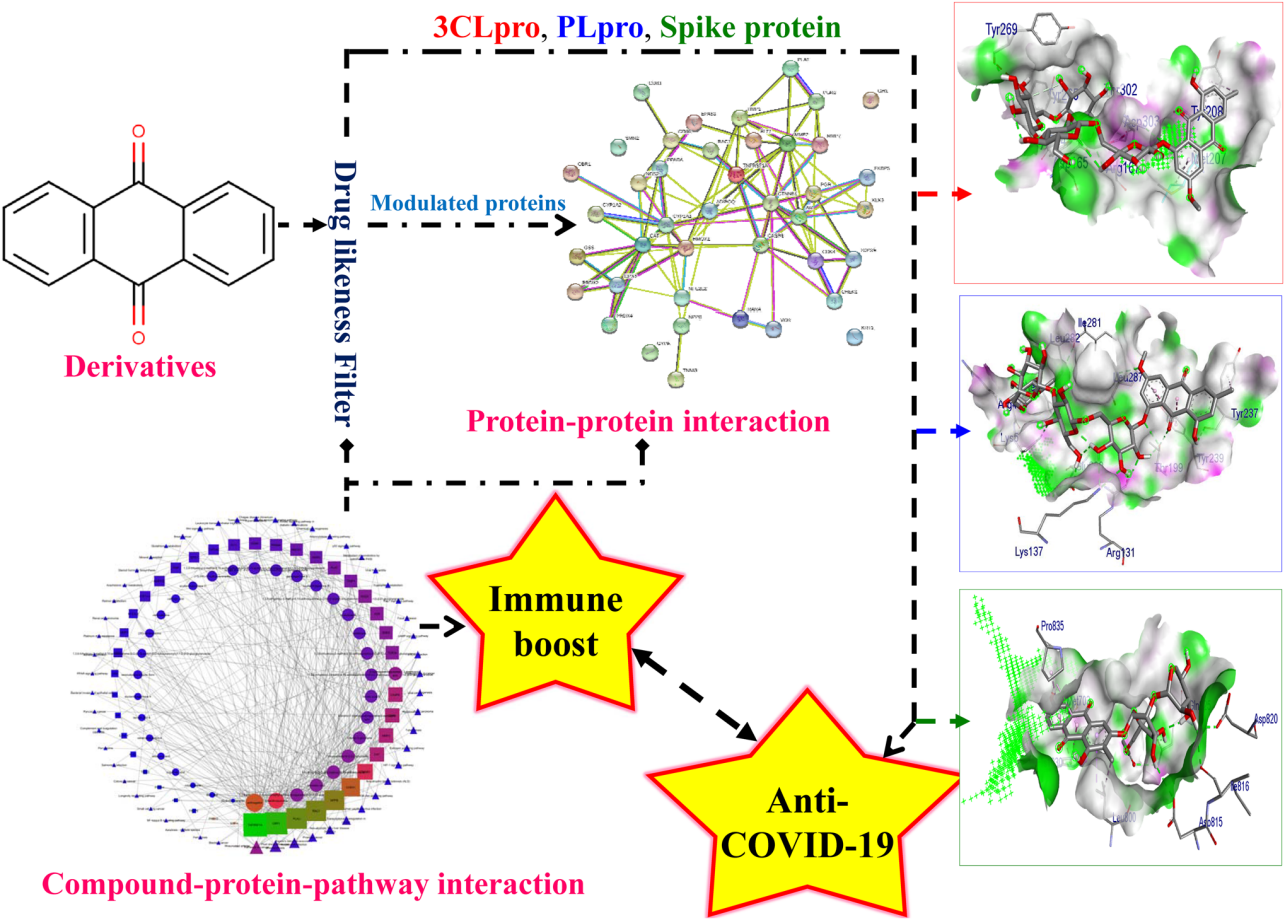

**Electronic supplementary material:**

The online version of this article (10.1007/s13659-020-00260-2) contains supplementary material, which is available to authorized users.

## Introduction

Presently, CoV Disease (COVID-19) has lead to millions of death throughout the world beginning from the December of 2019 [1]. Further, the risk of getting affected with COVID-19 is supplemented in subjects with lower immunity primarily special subjects like pediatrics/geriatrics and the patients suffering from infectious and non-infectious diseases [2]. Although approaches are being made to prevent this virus to get spread via social distancing and so on, boosting of immunity in subjects could play an important role in inhibiting the transmission of the virus and its invasion into the body. Although investigations are undergoing to develop the vaccine against COVID-19, it may still take time as the drug discovery process is much complicated. Hence, it is important to identify any alternative approach as prophylaxis against COVID-19 which can be implemented via the utilization of immune boosters from natural sources. Three targets of novel coronavirus i.e. 3C-like protease (3CLpro), papain-like protease (PLpro), and spike protein [3–6] are being targeted by multiple investigators to identify the new hit molecule for the management of COVID-19. Further, inflammation and cell necrosis are contributing factors in worsening the COVID-19 pathogenesis which suggests identifying the molecule with anti-viral, anti-inflammatory and immune-boosting properties.

Anthroquinolines are the group of compounds from multiple folk medicines like *Senna* species which are utilized in Ayurvedic system of medicines and Traditional Chinese Medicines for the management of various infectious and non-infectious diseases [7, 8]. Further, anthraquinone derivatives are also reported for anti-viral property [9], anti-inflammatory efficacy [10], and as immune booster [11]. Hence, in the COVID-19 infection, it may be beneficial if the bioactives are identified with an immune boost, anti-inflammatory, and anti-viral properties which can be demonstrated via the concept of network pharmacology or polypharmacological approach. Hence, based on the above theme, of anti-viral/anti-inflammatory/immune boosting reports of anthraquinones we attempted to screen the multiple anthraquinone derivatives as an immune booster and anti-viral efficacy using in silico molecular docking and other system biology tools.

## Materials and methods

### Bioactives and their Druglikeness Score

The reported phytoconstituents under the phytochemistry of anthraquinone were retrieved from available literature/Chemical Entities of Biological Interest (ChEBI) records (https://www.ebi.ac.uk/chebi/). All the compounds were then predicted for the druglikeness score by querying the SMILES of each molecule in MolSoft (https://molsoft.com/mprop/).

### Target Prediction and their Enrichment Analysis to Assess Immune-boosting Efficacy

Anthraquinone derivatives with positive druglikeness scores were queried in DIGEP-Pred [12] to identify “*Proteins based targets*” (up-regulated/downregulated proteins) at the probable activity of 0.5. The list of regulated proteins was queried in STRING [13] to identify the biological process, cellular function, and molecular processes of combined gene-set. Further, the probably modulated pathways were also identified concerning the Kyoto Encyclopedia of Genes and Genomes database. Network among the bioactives, their targets, and modulated pathways was constructed using the Cytoscape [14] version 3.5.1. Any duplicates interconnection of two nodes was eliminated to avoid the appearance of a false hit. The whole network was analyzed using the “*network analyzer*” tool based on node size and count representing the edge count as “*low values to small size*” and “*low values to bright colors*” respectively as explained previously [15].

### Prediction of Probable Anti-viral Activity

Anti-viral activity of each compound was predicted by querying the SMILES in Prediction of Activity Spectra for Substances [16] at the pharmacological activity (Pa) > pharmacological inactivity (Pi) and retrieving the probable biological spectrum for keyword “*anti-viral*”. Records were queried for their probable pharmacological spectrum against multiple viruses like Adenovirus, Cytomegalovirus (CMV), Hepatitis B, Hepatitis C, Hepatitis, Herpes, Human immunodeficiency virus (HIV), Influenza A, Influenza, Parainfluenza, Picornavirus, Poxvirus, Rhinovirus, and Trachoma.

### In Silico Molecular Docking

#### Preparation of Ligand Molecules

All the 3D. sdf format of ligand molecules with positive drug-likeness scores was retrieved from PubChem database (https://pubchem.ncbi.nlm.nih.gov/) or structures were drawn in ChemSketch (https://www.acdlabs.com/resources/freeware/chemsketch/) as applicable. The ligand molecules were converted into.pdb format using Discovery studio 2019 [17]. All the bioactives were minimized using MMFF94 forcefield [18] using conjugate gradients as an optimization algorithm. After the minimization of energy, all ligand molecules were converted into.pdbqt format.

#### Preparation of Macromolecules

Three target proteins of COVID-19 i.e. 3clpro (PDB: 6LU7), PLpro (PDB: 4M0W), and spike proteins (homology modeled target, accession number: AVP78042.1 as query sequence and PDB: 6VSB as a template using SWISS-MODEL [19]) were selected. The retrieved proteins from Research Collaboratory for Structural Bioinformatics database were in complex with other heteroatoms which were removed using Discovery studio 2019 and saved in.pdb format.

#### Ligand–protein Docking

The ligand molecules were docked with protein molecules using autodock4 [20] by setting 8 exhaustiveness as default to obtain 10 different poses of ligand molecules. After docking the pose of ligand scoring lowest binding energy was selected to visualize the ligand–protein interaction in Discovery Studio 2019 as explained previously [21, 22].

## Results

### Bioactives and their Druglikeness Score

The complete datasheet of 101 compounds including their name, ChEBI ID, molecular formula/weight, and synonym including phytochemistry for the retrieved compounds were summarized (Table S1). Among 101 different compounds, 36 were identified with positive druglikeness scores. Among them, laccaic acid A scored highest druglikeness score i.e. 0.85 with molecular weight 537.09, 12 hydrogen bond acceptor, 8 hydrogen bond donors, and 2.88 MolLogP. The details of the druglikeness score of each compound are summarized in Table 1.

**Table 1:** Druglikeness of anthraquinone derivatives with positive score

Bioactives	Molecular formula	Molecular weight	NHBA	NHBD	MolLogP	MolPSA (A^2^)	MolVol (A^3^)	DLS
Versicolorone tricyclic form	C_20_H_18_O_8_	386.1	8	5	1.67	122.82	375.84	0.09
(1′S,5′S)-5′-hydroxyaverantin	C_20_H_20_O_8_	388.12	8	6	1.9	125	371.14	0.17
(1′S,5′R)-5′-hydroxyaverantin	C_20_H_20_O_8_	388.12	8	6	1.9	125	371.14	0.17
chrysophanol 8-*O*-*β*-d-glucoside	C_21_H_20_O_9_	416.11	9	5	1.11	122.67	379.02	0.45
1,3,6-trihydroxy-2-methyl-9,10-anthraquinone-3-*O*-(6′-*O*-acetyl)-*α*-l-rhamnopyranosyl-(1- > 2)-*β*-d-glucopyranoside	C_29_H_32_O_15_	620.17	15	7	1.46	189.78	556.02	0.6
1,3,6-trihydroxy-2-methyl-9,10-anthraquinone-3-*O*-(6′-*O*-acetyl)-*β*-d-glucopyranoside	C_23_H_22_O_11_	474.12	11	5	2.07	143.34	436.83	0.45
1,3,6-trihydroxy-2-methyl-9,10-anthraquinone-3-*O*-*α*-l-rhamnopyranosyl-(1- > 2)-*β*-d-glucopyranoside	C_27_H_30_O_14_	578.16	14	8	0.85	185.66	510.27	0.5
1,3,6-trihydroxy-2-methyl-9,10-anthraquinone-3-*O*-(6′-*O*-acetyl)-*β*-d-xylopyranosyl-(1- > 2)-*β*-d-glucopyranoside	C_28_H_30_O_15_	606.16	15	7	1.01	190.6	540.52	0.69
1,3,6-trihydroxy-2-methyl-9,10-anthraquinone-3-*O*-(3′-*O*-acetyl)-*α*-l-rhamnopyranosyl-(1- > 2)-*β*-d-glucopyranoside	C_29_H_32_O_15_	620.17	15	7	1.42	190.3	556.1	0.76
1-hydroxy-2-(β-d-glucosyloxy)-9,10-anthraquinone	C_20_H_18_O_9_	402.1	9	5	1.1	121.6	358.41	0.04
(2S)-versicolorone	C_20_H_18_O_8_	386.1	8	5	1.67	122.82	375.84	0.09
(S)-5′-oxoaverantin	C_20_H_18_O_8_	386.1	8	5	1.77	121.86	374.32	0.16
BDA-366	C_24_H_29_N_3_O_4_	423.22	5	3	2.54	74.7	440.1	0.09
Emodin 8-glucoside	C_21_H_20_O_10_	432.11	10	6	0.61	140.29	389.71	0.74
Anthragallol	C_21_H_20_O_10_	432.11	10	6	0.61	140.29	389.71	0.74
Nogalonic acid	C_20_H_14_O_8_	382.07	8	3	2.33	113.17	370.61	0.44
Mitoxantrone	C_22_H_28_N_4_O_6_	444.2	8	8	-0.55	135.33	432.17	0.53
Torososide B	C_40_H_52_O_25_	932.28	25	14	-4.46	320.41	789.36	0.63
Versicolorone	C_20_H_16_O_7_	368.09	7	3	2.68	97.9	364.43	0.14
4′-*O*-demethylknipholone-4′-*O*-*β*-d-glucopyranoside	C_29_H_26_O_13_	582.14	13	8	1.91	184.8	529.73	0.61
Aklanonic acid	C_21_H_16_O_8_	396.08	8	3	2.83	112.59	389.73	0.51
Kermesic acid	C_16_H_10_O_8_	330.04	8	5	2.1	119.71	304.77	0.06
Gaboroquinone A	C_24_H_18_O_9_	450.1	9	5	3.35	130.76	428.49	0.27
Variecolorquinone A	C_20_H_18_O_9_	402.1	9	4	1.36	121.77	383.49	0.71
Laccaic acid A	C_26_H_19_NO_12_	537.09	12	8	2.88	186.68	496.66	0.85
Laccaic acid B	C_24_H_16_O_12_	496.06	12	8	3.19	178.91	446.68	0.65
Laccaic acid C	C_25_H_17_NO_13_	539.07	14	10	0.68	211.46	476.5	0.68
Carminic acid	C_22_H_20_O_13_	492.09	13	9	0.62	189.58	434.4	0.77
Ekatetrone	C_19_H_13_NO_7_	367.07	7	4	2.12	112.76	348.31	0.5
4-bromo-1-hydroxyanthraquinone-2-carboxylic acid	C_15_H_7_BrO_5_	345.95	5	2	4.39	70.07	269.12	0.24
Scutianthraquinone A	C_39_H_32_O_13_	708.18	13	4	7.75	165.64	711.03	0.45
Scutianthraquinone B	C_38_H_30_O_13_	694.17	13	4	7.41	165.64	693.71	0.26
Scutianthraquinone C	C_34_H_24_O_12_	624.13	12	5	5.88	161.07	618.22	0.31
1,3,6-trihydroxy-2-hydroxymethyl-9,10-anthraquinone-3-*O*-(6′-*O*-acetyl)-*β*-d-glucopyranoside	C_23_H_22_O_12_	490.11	12	6	0.93	160.45	445.01	0.41
Rubianthraquinone	C_16_H_12_O_5_	284.07	5	2	3.26	68.06	281.86	0.07
5-hydroxyanthraquinone-1,3-dicarboxylic acid	C_16_H_8_O_7_	312.03	7	3	3.47	99.54	281.87	0.28

*DLS* Druglikeness Score *NHBA* Number of Hydrogen Bond Acceptor *NHBD* Number of Hydrogen Bond Donor

### Target Prediction and their Enrichment Analysis to Assess Immune-Boosting Efficacy

**Fig. 1:**
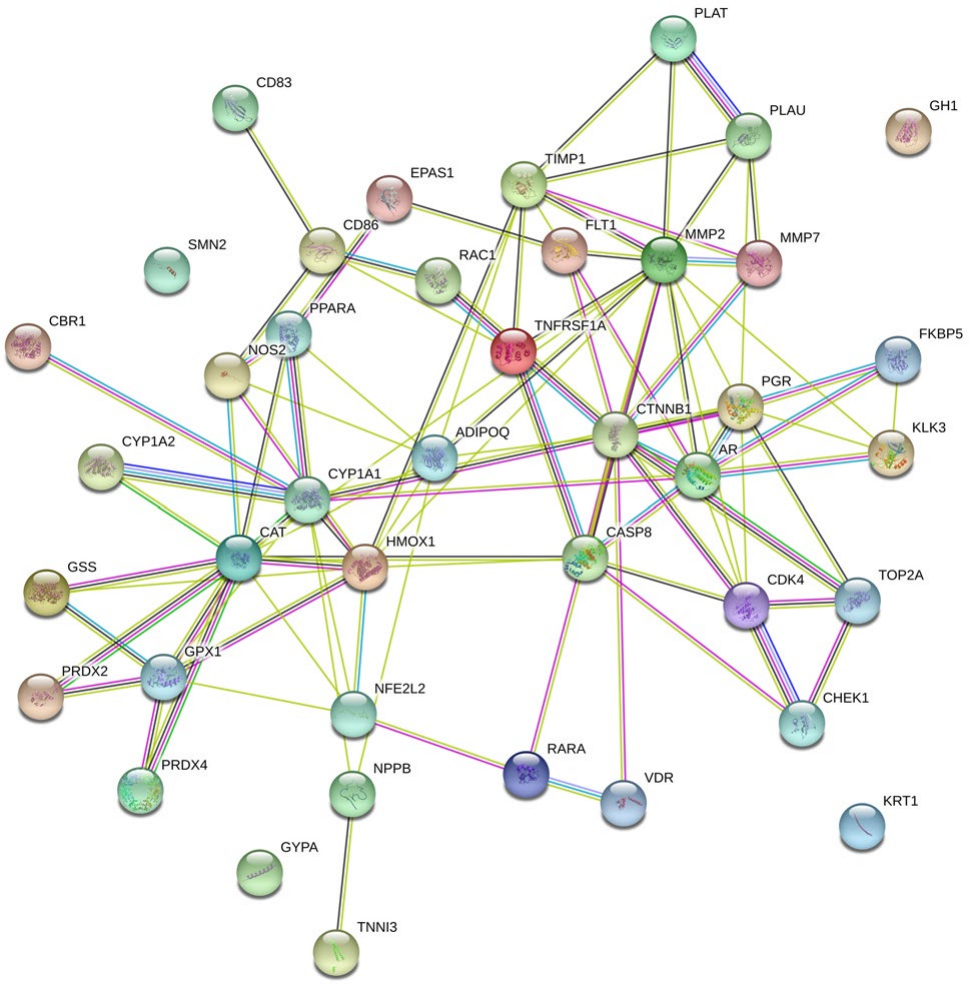
Protein–protein interaction of regulated proteins

**Fig. 2:**
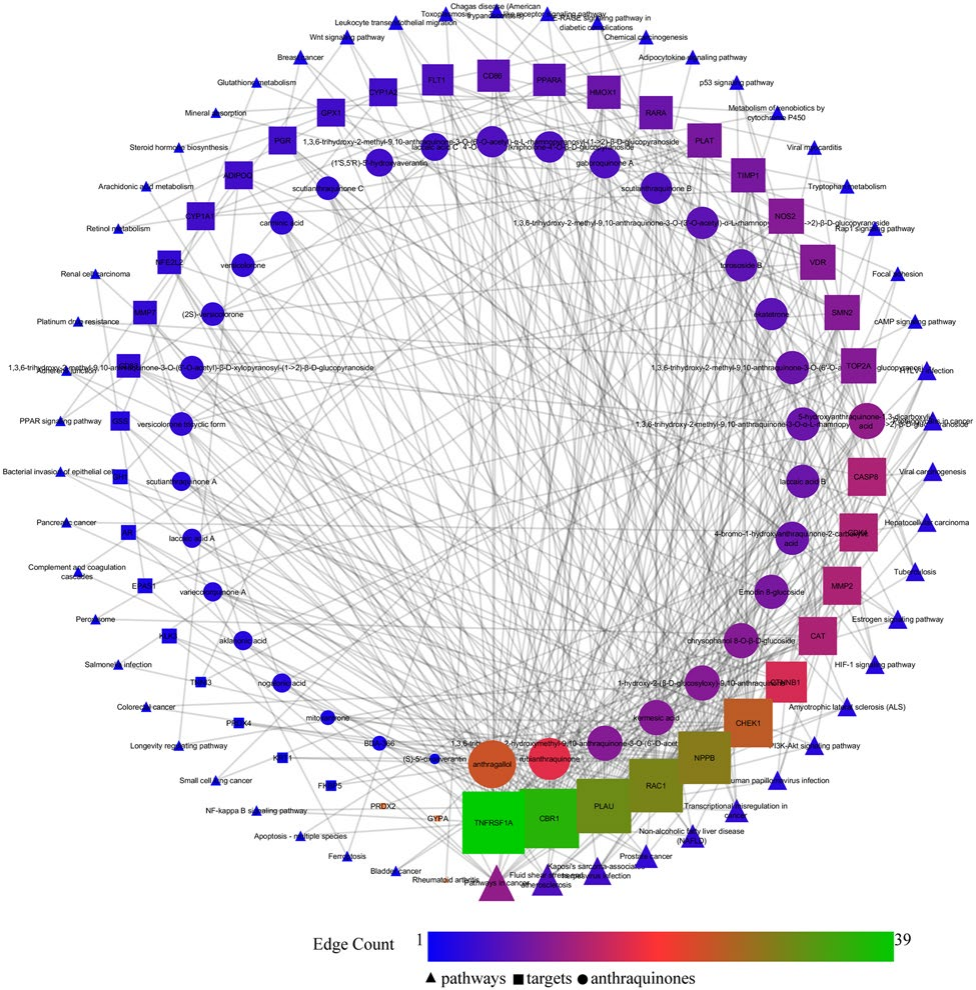
Network interaction of anthraquinone derivatives with their proteins and regulated pathways

Among the compounds with positive druglikeness score, anthragallol was predicted to modulate the highest number of genes i.e. 25. Similarly, human carbonyl reductase 1 (CBR1) was targeted by the highest number of bioactives i.e. 33. Further, the enrichment analysis identified the modulation of 54 different pathways in which pathways in cancer was majorly modulated by regulating 12 genes (AR, CASP8, CDK4, CTNNB1, EPAS1, HMOX1, KLK3, MMP2, NFE2L2, NOS2, RAC1, and RARA) under the background of 515 proteins at the false discovery rate of 7.71E−08. Table 2 summarizes the gene enrichment analysis of the modulated gene set along with modulated pathways with their respective gene codes. The protein–protein interaction of modulated proteins is presented in Fig. 1. Similarly, the combined bioactives-proteins-pathways also reflected the anthrogallol to target the highest number of proteins. Likewise, TNFRSF1A and pathways in cancer were majorly targeted/modulated protein and pathways respectively (Fig. 2).

**Table 2:** Enrichment analysis of modulated proteins by the reported anthraquinone derivatives

Term ID	Term description	Observed gene count	Background gene count	False discovery rate	Matching proteins in network
hsa05200	Pathways in cancer	12	515	7.71E−08	AR, CASP8, CDK4, CTNNB1, EPAS1, HMOX1, KLK3, MMP2, NFE2L2, NOS2, RAC1, RARA
hsa05418	Fluid shear stress and atherosclerosis	7	133	1.18E−06	CTNNB1, HMOX1, MMP2, NFE2L2, PLAT, RAC1, TNFRSF1A
hsa05167	Kaposi's sarcoma-associated herpesvirus infection	6	183	0.00012	CASP8, CD86, CDK4, CTNNB1, RAC1, TNFRSF1A
hsa05215	Prostate cancer	5	97	0.00012	AR, CTNNB1, KLK3, PLAT, PLAU
hsa05014	Amyotrophic lateral sclerosis (ALS)	4	50	0.00015	CAT, GPX1, RAC1, TNFRSF1A
hsa04932	Non-alcoholic fatty liver disease (NAFLD)	5	149	0.00044	ADIPOQ, CASP8, PPARA, RAC1, TNFRSF1A
hsa05202	Transcriptional misregulation in cancer	5	169	0.00068	CD86, FLT1, PLAT, PLAU, RARA
hsa04066	HIF-1 signaling pathway	4	98	0.0012	FLT1, HMOX1, NOS2, TIMP1
hsa00380	Tryptophan metabolism	3	40	0.0017	CAT, CYP1A1, CYP1A2
hsa04915	Estrogen signaling pathway	4	133	0.003	FKBP5, MMP2, PGR, RARA
hsa05416	Viral myocarditis	3	56	0.0037	CASP8, CD86, RAC1
hsa00980	Metabolism of xenobiotics by cytochrome P450	3	70	0.0053	CBR1, CYP1A1, CYP1A2
hsa04115	p53 signaling pathway	3	68	0.0053	CASP8, CDK4, CHEK1
hsa04920	Adipocytokine signaling pathway	3	69	0.0053	ADIPOQ, PPARA, TNFRSF1A
hsa05152	Tuberculosis	4	172	0.0053	CASP8, NOS2, TNFRSF1A, VDR
hsa05225	Hepatocellular carcinoma	4	163	0.0053	CDK4, CTNNB1, HMOX1, NFE2L2
hsa05203	Viral carcinogenesis	4	183	0.0056	CASP8, CDK4, CHEK1, RAC1
hsa05204	Chemical carcinogenesis	3	76	0.0056	CBR1, CYP1A1,CYP1A2
hsa05205	Proteoglycans in cancer	4	195	0.0065	CTNNB1, MMP2, PLAU, RAC1
hsa04933	AGE-RAGE signaling pathway in diabetic complications	3	98	0.0097	CDK4, MMP2, RAC1
hsa04620	Toll-like receptor signaling pathway	3	102	0.01	CASP8, CD86, RAC1
hsa05142	Chagas disease (American trypanosomiasis)	3	101	0.01	CASP8, NOS2, TNFRSF1A
hsa05145	Toxoplasmosis	3	109	0.0113	CASP8, NOS2, TNFRSF1A
hsa04670	Leukocyte transendothelial migration	3	112	0.0117	CTNNB1, MMP2, RAC1
hsa05166	HTLV-I infection	4	250	0.0121	CDK4, CHEK1, CTNNB1, TNFRSF1A
hsa04215	Apoptosis—multiple species	2	31	0.0131	CASP8, TNFRSF1A
hsa04216	Ferroptosis	2	40	0.0204	GSS, HMOX1
hsa04310	Wnt signaling pathway	3	143	0.0204	CTNNB1, MMP7, RAC1
hsa05219	Bladder cancer	2	41	0.0204	CDK4, MMP2
hsa05224	Breast cancer	3	147	0.0204	CDK4, CTNNB1, PGR
hsa05165	Human papillomavirus infection	4	317	0.0226	CASP8, CDK4, CTNNB1, TNFRSF1A
hsa00480	Glutathione metabolism	2	50	0.0261	GPX1, GSS
hsa04978	Mineral absorption	2	51	0.0263	HMOX1, VDR
hsa04151	PI3K-Akt signaling pathway	4	348	0.0285	CDK4, FLT1, GH1, RAC1
hsa00140	Steroid hormone biosynthesis	2	58	0.0316	CYP1A1, CYP1A2
hsa00590	Arachidonic acid metabolism	2	61	0.0337	CBR1, GPX1
hsa00830	Retinol metabolism	2	62	0.0338	CYP1A1, CYP1A2
hsa04024	cAMP signaling pathway	3	195	0.0339	PPARA, RAC1, TNNI3
hsa04510	Focal adhesion	3	197	0.034	CTNNB1, FLT1, RAC1
hsa04015	Rap1 signaling pathway	3	203	0.0359	CTNNB1, FLT1, RAC1
hsa05211	Renal cell carcinoma	2	68	0.0363	EPAS1, RAC1
hsa01524	Platinum drug resistance	2	70	0.0374	CASP8, TOP2A
hsa04520	Adherens junction	2	71	0.0375	CTNNB1, RAC1
hsa03320	PPAR signaling pathway	2	72	0.0376	ADIPOQ, PPARA
hsa05100	Bacterial invasion of epithelial cells	2	72	0.0376	CTNNB1, RAC1
hsa05212	Pancreatic cancer	2	74	0.0379	CDK4, RAC1
hsa04610	Complement and coagulation cascades	2	78	0.0409	PLAT, PLAU
hsa04146	Peroxisome	2	81	0.0429	CAT, NOS2
hsa05132	Salmonella infection	2	84	0.045	NOS2, RAC1
hsa05210	Colorectal cancer	2	85	0.045	CTNNB1, RAC1
hsa05323	Rheumatoid arthritis	2	84	0.045	CD86, FLT1
hsa04211	Longevity regulating pathway	2	88	0.0462	ADIPOQ, CAT
hsa05222	Small cell lung cancer	2	92	0.0492	CDK4, NOS2
hsa04064	NF-kappa B signaling pathway	2	93	0.0493	PLAU, TNFRSF1A

### Prediction of Probable Anti-viral Activity

**Fig. 3:**
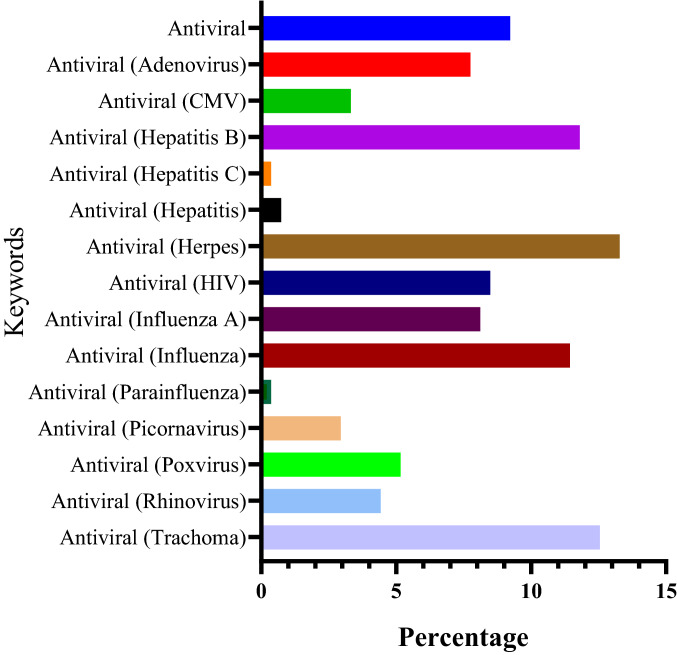
Predicted anti-viral activity of anthraquinone derivatives against multiple viruses

The anthraquinones were found to be anti-viral agents against adenovirus, CMV, hepatitis B and C, hepatitis, herpes, HIV, influenza A, influenza, parainfluenza, picornavirus, poxvirus, rhinovirus, and trachoma. Among them, the majority of the compounds were active against herpes virus i.e. 13.28%. The overall activity of compounds against multiple viruses is summarized in Fig. 3.

### In Silico Molecular Docking

**Fig. 4:**
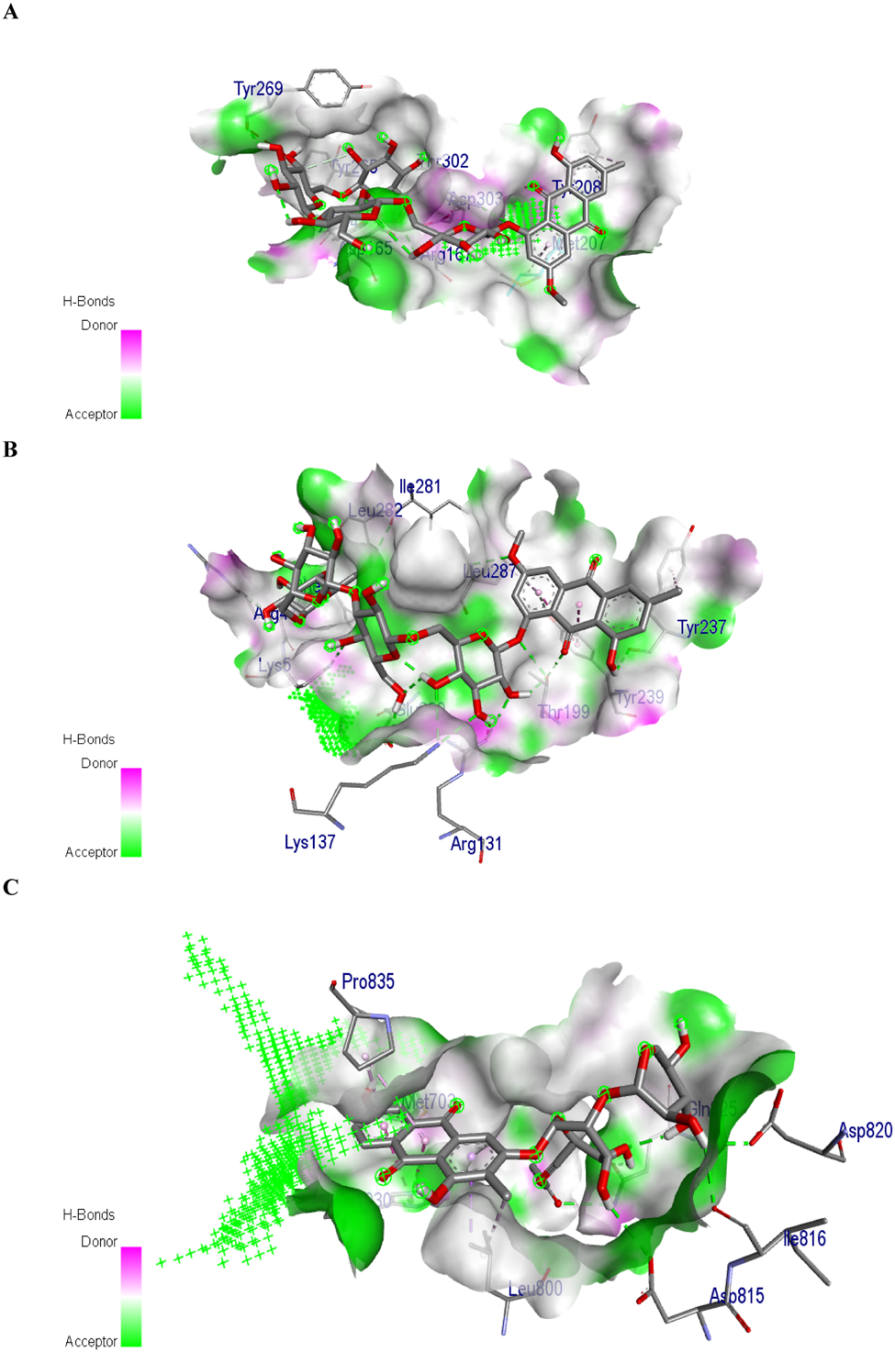
Interaction of torososide B with (**a**) Papain-like protease and (**b**) coronavirus main proteinase and 1,3,6-trihydroxy-2-methyl-9,10-anthraquinone-3-*O*-(6′-*O*-acetyl)-*β*-d-xylopyranosyl-(1→ 2)-*β*-d-glucopyranoside (**c**) with spike protein

Torososide B was predicted to have the highest binding affinity (− 8.7 kcal/mol) with PLpro with 9 hydrogen bond interactions via THR302, ASP303, TYR274, TYR265, ARG167, TYR269, ASP165. Further, Torososide B was predicted to possess the highest binding affinity (− 9.3 kcal/mol) with 3CLpro with 14 hydrogen bond interactions with LEU287, TYR237, THR199, ARG131, LYS137, LYS5, GLU290, ILE281, LEU282, and PHE3. Similarly, 1,3,6-trihydroxy-2-methyl-9,10-anthraquinone-3-*O*-(6′-*O*-acetyl)-*β*-d-xylopyranosyl-(1– > 2)-*β*-d-glucopyranoside was predicted to possess the highest binding affinity (− 8.7 kcal/mol) with spike protein with the highest number of hydrogen bond interactions i.e. 6 with ASP820, ILE816, ASP815, GLN825, and MET703. The binding affinity of each compound with individual targets with the number of hydrogen bond interactions and residues is summarized in Table S2. The interaction of Torososide B with PLpro and 3CLpro and 1,3,6-trihydroxy-2-methyl-9,10-anthraquinone-3-*O*-(6′-*O*-acetyl)-*β*-d-xylopyranosyl-(1– > 2)-*β*-d-glucopyranoside with spike protein is presented in Fig. 4.

## Discussion

During the COVID-19 infection, severe necrosis and inflammation lead to defects in the supply of necessary nutrients and oxygen into the cells which are more terrible in the subjects with compromised immunity. Hence, in the present study, we investigated multiple anthraquinone derivatives from various traditional medicines to act against COVID-19 targets i.e. 3CLpro, PLpro, and spike protein, and their combined immune-boosting efficacy. Initially, we calculated the druglikeness score of each molecule based on the “*Lipinksi’s Rule of Five*” [23] as the majority of the plant-based medicines are utilized via the oral route which identified 36 different compounds with positive druglikeness score and considered to get absorbed orally (Table 1) which were contemplated for further study.

The conventional drug discovery process utilizes the concept of “*single drug-single protein-single disease*” [24] which may not be applicable in the management of infectious diseases. This is due to the affinity of the pathogens (viruses/bacteria) to affect the multiple homeostatic functions of protein molecules. It means multiple proteins from the pathogens are involved to generate this effect. Hence, this can be managed via the utilization of modified drug development process “*multi compound-multi protein-disease*” interaction in which multiple bioactives regulate multiple proteins [25] which can also be taken as a basic key of boosting the immune system. Hence, in the present study, the combined synergistic phenomena of anthraquinones were investigated rather than a single bioactive molecule to identify multiple pathways that are directly or indirectly involved in the immune system.

Gene set enrichment analysis identified multiple pathways like p53 signaling pathway [26], PI3K-Akt signaling pathway [27], Rap1 signaling pathway [28], NF-kappa B signaling pathway [29] which are directly involved in the boosting the immune system. Similarly, some other pathways like pathways in cancer, PPAR signaling pathway, colorectal cancer, chemical carcinogenesis, estrogen signaling pathway were also identified which reflects the potency of anthraquinones to be beneficial in the subjects which are suffering from these pathways associated diseases like cancer. Further, pathways like p53 signaling pathways, PI3K-Akt, Wnt signaling pathways are also associated with diseases like diabetes and obesity where the immune system is compromised. Hence, regulation of these pathways could be beneficial in managing the diseases from which they are suffering, and boosting the immune system will also act as prophylaxis against COVID-19. Additionally, the enrichment analysis also identified the modulation of multiple pathways that are associated with a pathogenic infection like viral myocarditis and tuberculosis which reflects the potency of anthraquinone derivatives to manage the infectious diseases. Further, herbal medicines rich in anthraquinones also possess the anti-viral potency against various viruses. Hence, we attempted to identify the probable anti-viral activity of the anthraquinones with positive druglikeness score which identified their efficacy against multiple viruses like the rhino, influenza, herpes trachoma, pox, and CMV.

3CLpro alters the ubiquitin system to incorporate the viral polypeptides and deregulates the homeostatic task of functional proteins [30] which was majorly targeted by torososide B. Further, PLpro alters the function of protein phosphatase 1A and protein phosphatase 1B into the replicase proteins to adjust viral life cycle [31] which was majorly inhibited by torososide B. Similarly, spike protein utilizes angiotensin-converting enzyme 2 (ACE-2) as a receptor to enter inside the host cell [32, 33] which was chiefly regulated by modulated by 1,3,6-trihydroxy-2-methyl-9,10-anthraquinone-3-*O*-(6′-*O*-acetyl)-*β*-d-xylopyranosyl-(1→ 2)-*β*-d-glucopyranoside. These results reflect the probability of the anthraquinone derivatives to act as the anti-viral against COVID-19. However, as the time proceeds, it is to be understood that the binding affinity of probable lead hit molecules may get altered due to mutation in the possible protein targets and the inhibitory function may not occur as predicted.

## Conclusion

The present study utilized in silico molecular docking tools to identify the binding affinity of previously recorded anthraquinones derivatives against 3clpro, PLpro, and spike protein which identified Torososide B and 1,3,6-trihydroxy-2-methyl-9,10-anthraquinone-3-*O*-(6′-*O*-acetyl)-*β*-d-xylopyranosyl-(1→ 2)-*β*-d-glucopyranoside as a lead hits. Similarly, the combined synergies of the network identified the modulation of multiple pathways involved in the immune system like p53, chemokine, and PI3K-Akt signaling pathways. Additionally, anthraquinone derivatives were also identified as the modulators of the disease pathways where the immune system is compromised like diabetes and obesity. All these results suggest the probable therapeutic option of the utilization of anthraquinones as an immune booster and anti-viral against novel coronavirus by acting on the three targets as investigated. However, the present findings are only based on the computer simulations which need to be further validated using well designed experimental protocols.

## Electronic supplementary material

Below is the link to the electronic supplementary material.

Supplementary file1 (XLSX 54 kb)
